# Ubiquitin-specific protease 12 interacting partners Uaf-1 and WDR20 are potential therapeutic targets in prostate cancer

**DOI:** 10.18632/oncotarget.6075

**Published:** 2015-10-10

**Authors:** Urszula L. McClurg, Victoria J. Harle, Arash Nabbi, Amanda Batalha-Pereira, Scott Walker, Kelly Coffey, Luke Gaughan, Stuart RC McCracken, Craig N. Robson

**Affiliations:** ^1^ Solid Tumour Target Discovery Laboratory, Newcastle Cancer Centre, Northern Institute for Cancer Research, Medical School, Newcastle University, Newcastle upon Tyne, United Kingdom; ^2^ Department of Biochemistry and Molecular Biology, Southern Alberta Cancer Research Institute, Cumming School of Medicine, University of Calgary, Calgary, AB, Canada

**Keywords:** androgen receptor, deubiquitination, prostate cancer, Usp12, UAF1

## Abstract

The androgen receptor (AR) is a key transcription factor in the initiation and progression of prostate cancer (PC) and is a major therapeutic target for the treatment of advanced disease. Unfortunately, current therapies are not curative for castration resistant PC and a better understanding of AR regulation could identify novel therapeutic targets and biomarkers to aid treatment of this disease. The AR is known to be regulated by a number of post-translational modifications and we have recently identified the deubiquitinating enzyme Usp12 as a positive regulator of AR. We determined that Usp12 deubiquitinates the AR resulting in elevated receptor stability and activity. Furthermore, Usp12 silencing was shown to reduce proliferation of PC cells.

Usp12 is known to require the co-factors Uaf-1 and WDR20 for catalytic activity. In this report we focus further on the role of Uaf-1 and WDR20 in Usp12 regulation and investigate if these co-factors are also required for controlling AR activity. Firstly, we confirm the presence of the Usp12/Uaf-1/WDR20 complex in PC cells and demonstrate the importance of Uaf-1 and WDR20 for Usp12 stabilisation. Consequently, we show that individual silencing of either Uaf-1 or WDR20 is sufficient to abrogate the activity of the Usp12 complex and down-regulate AR-mediated transcription via receptor destabilisation resulting in increased apoptosis and decreased colony forming ability of PC cells. Moreover, expression of both Uaf-1 and WDR20 is higher in PC tissue compared to benign controls. Overall these results highlight the potential importance of the Usp12/Uaf-1/WDR20 complex in AR regulation and PC progression.

Highlights:

- Androgen receptor is a key transcriptional regulator in prostate cancer
- Usp12/Uaf-1/WDR20 complex plays a crucial role in androgen receptor stability and activity
- Destabilising an individual Usp12/Uaf-1/WDR20 complex member reduces the protein levels of the whole complex and diminishes androgen receptor activity
- Protein levels of all members of the Usp12/Uaf-1/WDR20 complex are significantly increased in PC

## INTRODUCTION

Prostate cancer (PC) is the most commonly diagnosed male malignancy in the United States [1]. The androgen receptor (AR) is a nuclear hormone receptor with a key role in regulating the transcription of genes required for PC growth and progression, therefore the AR axis is a major focus of current therapies for advanced PC [2]. Unfortunately, despite an initial response these approaches invariably fail due to aberrant AR signalling mediated by receptor mutations and splice variants, AR amplification and ligand-independent AR activation [3] resulting in the development of castrate-resistant prostate cancer (CRPC). The current lack of curative therapy for late stage PC highlights the need for both new therapeutic targets to overcome resistance as well as novel biomarkers to aid early identification of PC and allow better patient stratification.

The AR itself is subject to tight regulation by a series of post-translational modifications, including acetylation, methylation, phosphorylation, sumoylation and ubiquitination [4]; [5]; [6]; [7]; [8]; [9]; [10]. Proteins involved in the upstream regulation of the AR are therefore interesting therapeutic targets as targeting them could provide an additional therapeutic opportunity for CRPC [11]. We have recently identified the deubiquitinase (DUB), ubiquitin specific protease 12 (Usp12) as a positive regulator of the AR responsible for deubiquitinating and subsequently stabilising the receptor. Usp12 acts as an AR co-activator both directly by deubiquitinating the AR [12] and indirectly by regulating AR-AKT cross-talk [13]. We further established that Usp12 depletion reduced PC cell proliferation and induced apoptosis, furthermore Usp12 levels are increased in PC tissue [12].

Usp12 is a highly conserved protein with a high degree of homology to Usp46 and to a lesser extent Usp1. To date the only other reported targets of Usp12 are histones H2A and H2B [14], non-activated Notch [15] and the phosphatases PHLPP and PHLPPL [13]. Usp1-associated factor 1 (Uaf-1) is a WD40 repeat (WDR) containing protein essential for Usp12 activity, this complex is additionally further stabilised by WDR20 [16-18].

In this paper we have further interrogated how the Usp12 complex functions and investigated the effects of depleting Uaf-1 or WDR20 on Usp12 activity and subsequent destabilisation of the AR. The rationale behind this is that due to high homology within the catalytic domains of DUBs it is predicted that developing selective drugs is likely to be problematic [19]. Therefore, targeting DUB cofactors such as Uaf-1 and WDR20 directly or targeting DUB-cofactor interactions may allow the development of more targeted therapeutics [11]. This has already been evidenced by the development of therapeutics aimed at the Usp1-Uaf-1 complex, which have been shown to reverse cisplatin-resistance in non-small lung cancer cells [20, 21].

We have further investigated the regulation of Usp12 by Uaf-1 and WDR20 and shown that these two co-factors stabilise Usp12 at the protein level. Depletion of any of the complex members affects the transcript levels of the Usp12 binding partners. We have established an essential role for the Uaf-1 and WDR20 proteins in AR regulation and demonstrated that a reduction in either one of these proteins reduces AR stability, transcriptional activity and importantly PC cell survival. We have also determined that Uaf-1 and WDR20 are both overexpressed in PC tissue indicating that they could be promising PC biomarkers. Overall, this work further highlights the potential of the Usp12 complex as a potential therapeutic drug target in PC.

## RESULTS

### Usp12, Uaf-1 and WDR20 form a complex acting within a positive feedback loop in PC cells

**Figure 1:**
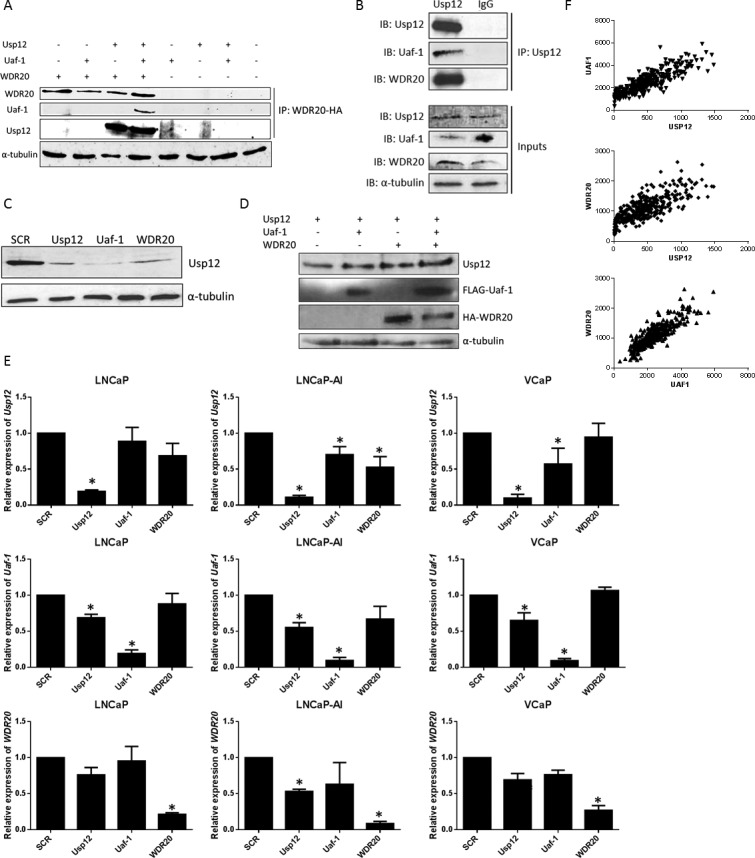
Usp12, Uaf-1 and WDR20 form a complex acting within a positive feedback loop **A.** HEK293T cells transfected as indicated and incubated for 48 h prior to lysis. Exogenous WDR20 was immunoprecipitated using 1 μg anti-HA antibody and samples analysed by immunoblotting. **B.** Endogenous Usp12 was immunoprecipitated from LNCaP cells and samples probed for the presence of Uaf-1 and WDR20 by immunoblotting. IgG antibody was used as a negative control. **C.** LNCaP cells were subject to 96 h siRNA silencing as indicated followed by lysis and immunoblotting. **D.** pFlag-Usp12, pFlag-Uaf-1 and pHA-Flag-WDR20 were overexpressed in HEK293T cells as indicated. Cells were incubated for 48 h prior to lysis and immunoblotted. **E.** LNCaP, LNCaP-AI or VCaP cells were treated with siRNA for 96 h as indicated prior to RNA extraction and quantitative qRT-PCR. All data is normalised to expression of the control gene HPRT1 and displayed as expression relative to scrambled (SCR). Error bars represent standard error of the means (SEM) of three independent experiments. **F.** Comparison of *Usp12*, *Uaf-1* and *WDR20* gene expression counts from the TCGA RNA sequencing data in a prostate cancer dataset (*n* = 340).

Usp12 is known to form a complex with Uaf-1 and WDR20 [16, 17]. To confirm the formation of this complex WDR20, Uaf-1 and Usp12 were overexpressed followed by exogenous WDR20 immunoprecipitation. Subsequent immunoblotting confirmed the interaction between these three proteins and interestingly revealed that Uaf-1 does not directly interact with WDR20 in the absence of Usp12 (Figure 1A). This further confirms comprehensive analysis by Kee *et al*. who established that WDR20 binds with higher affinity to the complex than to the Usp12 or Uaf-1 alone [17]. To establish if this complex is present in PC cells, Usp12 was immunoprecipitated from the PC LNCaP cell line. Results confirmed the interaction between endogenous Usp12, Uaf-1 and WDR20 in PC cells (Figure 1B).

Additionally, we observed that Usp12 protein levels were consistently higher when both Uaf-1 and WDR20 were present. Uaf-1 and WDR20 have previously been shown to stimulate Usp12 catalytic activity [17, 18]. To determine if these additionally affect Usp12 protein stability Uaf-1 and WDR20 were silenced in LNCaP cells. Depletion of either complex member reduced Usp12 protein levels (Figure 1C). To confirm our findings Usp12 was overexpressed either alone or in combination with Uaf-1 and WDR20. As predicted Usp12 levels were stabilised by the presence of its cofactors (Figure 1D).

To determine if this stabilisation is due to regulation at a transcriptional level, mRNA was quantified following depletion of each complex member in three different PC cell lines. We used LNCaP as a model of androgen sensitive disease, LNCaP-AI as a model of androgen independent PC and VCaP as a model of AR amplified disease with AR variants. Reduction of Uaf-1 diminished the levels of *Usp12* transcripts in the LNCaP-AI and VCaP cell lines (Figure 1E). Similarly, Usp12 depletion reduced both *Uaf-1* and *WDR20* at an mRNA level. Overall, suggesting that this complex may act within a feedback loop. This result was further confirmed in patient data. We analysed the TCGA database of RNA-seq data and observed a significant correlation (p>0.0001 in all three cases) between the Usp12, Uaf-1 and WDR20 gene expression in PC patient samples (Figure 1F). Additionally, ZODIAC analysis [22] of the Usp12 complex copy number, gene expression and methylation status in TCGA database revealed that Usp12 gene expression levels are significantly positively correlated with Uaf-1 and WDR20 gene expression across all of TCGA sample datasets and additionally a positive correlation between Usp12 and Uaf-1 methylation was observed (sup fig. 1).

### Uaf-1 and WDR20 interact with and stabilise the AR

**Figure 2:**
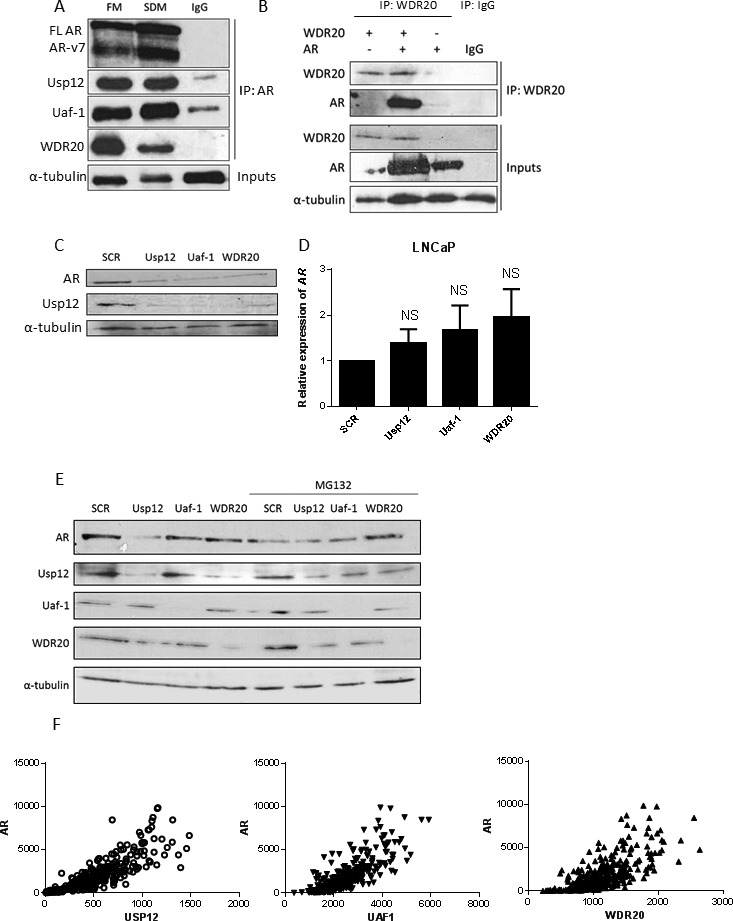
*Uaf-1* and *WDR20* form a complex with AR resulting in AR protein stabilisation **A.** VCaP cells were cultured in full media (FM) or steroid depleted media (SDM) for 96 h prior to lysis. Endogenous AR was immunoprecipitated using 1 μg anti-AR antibody or a negative IgG control. Samples were analysed by immunoblotting with both AR isoforms visible (FL AR - full length AR, ARv7- AR isoform 7 consisting of exons 1, 2, 3 and cryptic exon 3 [45]). **B.** COS-7 cells were transfected with pFlag-AR or pHA-Flag-WDR20 plasmids as indicated. 48 h later cells were harvested and subjected to immunoprecipitation for WDR20 or an IgG control followed by immunoblotting. **C.** LNCaP cells subject to 96 h siRNA knockdown as indicated prior to lysis and immunoblotting. **D.** LNCaP cells were treated with siRNA for 96 h followed by RNA extraction and qRT-PCR analysis normalised to HPRT1 levels. Data is presented as a mean of three independent experiments +/− SEM with p values compared to scrambled treated control. **E.** LNCaP cells were subject to 96 h siRNA silencing as indicated followed by lysis and immunoblotting. Where indicated cells were treated with MG132 for 8h prior to lysis. **F.** Comparison of ***Usp12***, ***Uaf-1*** or *WDR20* gene expression versus *AR* from the TCGA prostate cancer dataset (*n* = 340).

We have previously established that AR and Usp12 interact [12]. As both Uaf-1 and WDR20 interact with Usp12 we hypothesised that Uaf-1 and WDR20 would also be found in a complex with AR. Uaf-1 and WDR20 were shown to interact with AR and Usp12 endogenously in the VCaP cell line (Figure 2A), confirming the presence of this complex in PC cells. To assess if WDR20 can interact with AR we overexpressed both proteins in HEK293T cells. Similarly, we determined that WDR20 is found in a complex with AR (Figure 2B).

We have previously demonstrated that Usp12 is an AR coactivator [12]. Given the role of Uaf-1 and WDR20 in stabilisation and activation of Usp12 we hypothesised that depletion of these proteins alone should be sufficient to reduce AR protein levels. To investigate this we examined AR levels in LNCaP cells following depletion of Uaf-1 or WDR20. Importantly, depletion of either protein reduced AR levels potentially as a consequence of reduction in Usp12 (Figure 2C). This change in AR levels is post-translational as depletion of Usp12, Uaf-1 or WDR20 had no significant effect on AR transcript levels (Figure 2D). We have confirmed that this effect on AR protein stability was through the ubiquitin proteasome system as upon MG132 treatment Usp12, Uaf-1 or WDR20 depletion had no effect on AR protein stability (Figure 2E). Interestingly even after the MG132 treatment individual complex members were still required for the protein stability of the Usp12 complex implying that this stabilisation does not occur via deubiquitination (Figure 2E).

Our observation was further confirmed in patient data when we compared the *Usp12*, *Uaf-1* and *WDR20* gene expression versus that of AR from the TCGA database. Counts for all three members of the Usp12 complex were significantly correlated (p<0.0001) with the *AR* (Figure 2F). The effects of Usp12 complex members depletion on AR suggest that targeting Uaf-1 or WDR20 individually may be as effective as targeting Usp12 directly while it might provide higher specificity as proved in the case of Usp1/Uaf-1 targeting therapeutics.

### Uaf-1 and WDR20 are involved in the regulation of AR activity

**Figure 3:**
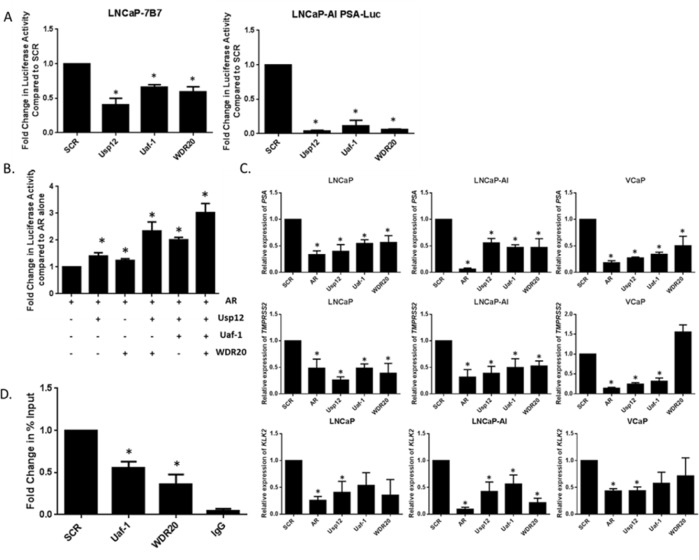
Uaf-1 and WDR20 are involved in the regulation of AR transcriptional activity **A.** Luciferase assay conducted in LNCaP-7b7 or LNCaP-AI PSA-Luc cells. Cells were subject to 96 h siRNA treatment in their respective growth media. After 96 h cells were imaged at a single time point using IncuCyte to determine cell density. Cells were lysed and luciferase assay conducted. Luciferase activity was normalised to cell density to correct for the effect of siRNA treatment on cellular proliferation. Data is displayed as fold change compared to SCR. Error bars represent SEM and results are a mean of three independent experiments. **B.** Luciferase assay conducted in HEK293T cells. Cells were starved in steroid-depleted conditions for 24 h prior to transfection with pARE-Luc, pβ-galactosidase and additional plasmids as indicated. Cells were incubated for further 24 h before stimulation with 10 nM DHT for the final 24 h. Luciferase activity was normalised to β-galactosidase activity to correct for transfection efficiency. Results are displayed as fold change normalised to AR overexpression alone. Error bars represent SEM and results are a mean of three independent experiments. **C.** LNCaP, LNCaP-AI or VCaP cells subject to 96 h siRNA treatment as indicated prior to RNA extraction and quantitative qRT-PCR. Data is normalised to HPRT1 and displayed as expression relative to SCR. Error bars represent standard error of the mean (SEM) and results are a mean of three independent experiments. **D.** ChIP analysis of AR recruitment at AREIII promoter of *PSA*. LNCaP cells were subject to 96 h siRNA knockdown under steroid-depleted conditions and stimulated with 10 nM DHT 2 h prior to chromatin extraction. ChIP was conducted for AR or a negative IgG control. Error bars represent SEM and results are a mean of three independent experiments.

As Uaf-1 and WDR20 silencing alone was sufficient to reduce the AR protein levels we investigated if it would affect AR activity. Firstly, luciferase reporter assays were conducted following Uaf-1 and WDR20 silencing in the LNCaP variant cell line LNCaP-7B7. These cells have a stably integrated *luciferase* gene located downstream of the androgen-responsive element of the *PSA* promoter. We have previously used this method to determine that Usp12 acts as an AR co-activator; Usp12 and AR were therefore included as positive controls. Depletion of WDR20, and to a lesser extent Uaf-1, reduced AR transcriptional activity (Figure 3A). This assay was repeated using a variant of the LNCaP-AI cell line generated in the same manner. In this cell line silencing of each complex member resulted in a dramatic decrease in AR activity (Figure 3A).

These findings were confirmed by transfecting cells with a reporter containing three adjacent androgen-responsive elements (AREs) upstream from the *luciferase* gene, alongside AR, Usp12, Uaf-1 and WDR20 and β-galactosidase for normalisation. Cells were subsequently stimulated with dihydrotestosterone (DHT) and receptor activity assessed. We have previously demonstrated that Usp12/Uaf-1 complex increases AR transcriptional activity. Here we demonstrate the importance of WDR20 in this process. Interestingly, Usp12/WDR20 alone significantly increased AR activity but this effect was greatest when Usp12, Uaf-1 and WDR20 were overexpressed simultaneously (Figure 3B). Cell lines used in this study express low levels of endogenous Usp12 and Uaf-1, consequently even without the addition of Uaf-1 overexpression of WDR20 alone was enough to significantly enhance the AR activity. This is a result of enhanced activity of the endogenous Usp12 complex caused by increased levels of WDR20 which supports our data on the positive feedback loop within this complex. This further confirms that each complex member alone has a limited effect on AR activity but in combination the Usp12 complex significantly increases AR transcriptional activity implying that targeting any of the binding partners should offer the same efficacy as targeting Usp12 alone.

To further validate our results Usp12, Uaf-1 and WDR20 were silenced in three different PC cell lines and transcript levels of the AR regulated genes *PSA, TMPRSS2* and *KLK2* determined. Silencing of each complex member reduced the transcript levels of all AR target genes in LNCaP and LNCaP-AI cells with similar effects observed in VCaPs (Figure 3C). Finally, we used chromatin immunoprecipitations to show that silencing of Uaf-1 or WDR20 reduced AR recruitment to AREIII of *PSA* (Figure 3D).

Overall these results confirm our hypothesis that reduction of Uaf-1 or WDR20 alone is sufficient to reduce AR activity. The similarity in the reduction of AR activity between the cell lines is particularly important as it implies that Usp12 complex is a regulator of AR in all stages of PC and as such could be a valid therapeutic target in CRPC.

### Uaf-1 and WDR20 are involved in PC survival and proliferation

**Figure 4:**
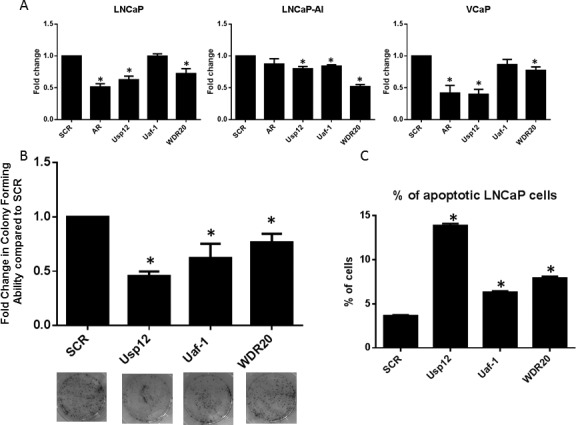
Uaf-1 and WDR20 regulate PC survival and proliferation **A.** LNCaP, LNCaP-AI or VCaP cells were treated with siRNA for 96 h as indicated. Cells were cultured in their respective growth media prior to cell counting. Cell count is displayed as fold change compared to SCR. Error bars represent SEM and data is a mean of three independent experiments. **B.** LNCaP cells treated with siRNA for 72 h were seeded at equal densities in 6 well plates. Cells were incubated for 14 days prior to staining with crystal violet. Colonies were counted and results normalised to SCR. Error bars represent SEM and results are a mean of three independent experiments. Representative pictures of the wells are displayed below. **C.** LNCaP cells subject to 96 h siRNA silencing as indicated. Flow cytometry was conducted to detect annexin-V and PI positive cells. Results are normalised to SCR and results are a mean of three independent experiments +/− SEM.

To establish if Uaf-1 and WDR20 have a role in PC proliferation both complex members were silenced alongside Usp12 and AR in different cell line models of PC. Proliferation rate was subsequently determined by cell counting. Depletion of WDR20 reduced proliferation in all tested cell lines, whilst Uaf-1 silencing significantly reduced proliferation in the LNCaP-AI cell line (Figure 4A). Moreover, the ability of LNCaP cells to form colonies was significantly reduced following Uaf-1 or WDR20 depletion (Figure 4B).

The effect of Uaf-1 and WDR20 depletion on cell survival was assessed using Annexin V staining to detect apoptotic cells. Staining was quantified using flow cytometry and we observed that Uaf-1 and WDR20 silencing alone significantly increased the levels of apoptotic cells (Figure 4C). These results confirm that silencing of either Uaf-1 or WDR20 alone is sufficient to reduce PC cell proliferation, induce apoptosis and reduce colony forming ability. Overall, indicating that silencing of the cofactors required for Usp12 activity can be as efficient in affecting PC cell growth and survival as targeting Usp12 directly. Again the observation that silencing of individual complex members can reduce proliferation in PC cell lines representing different stages of the disease highlights the potential importance of this complex in CRPC.

### Uaf-1 and WDR20 are biomarkers of PC

**Figure 5:**
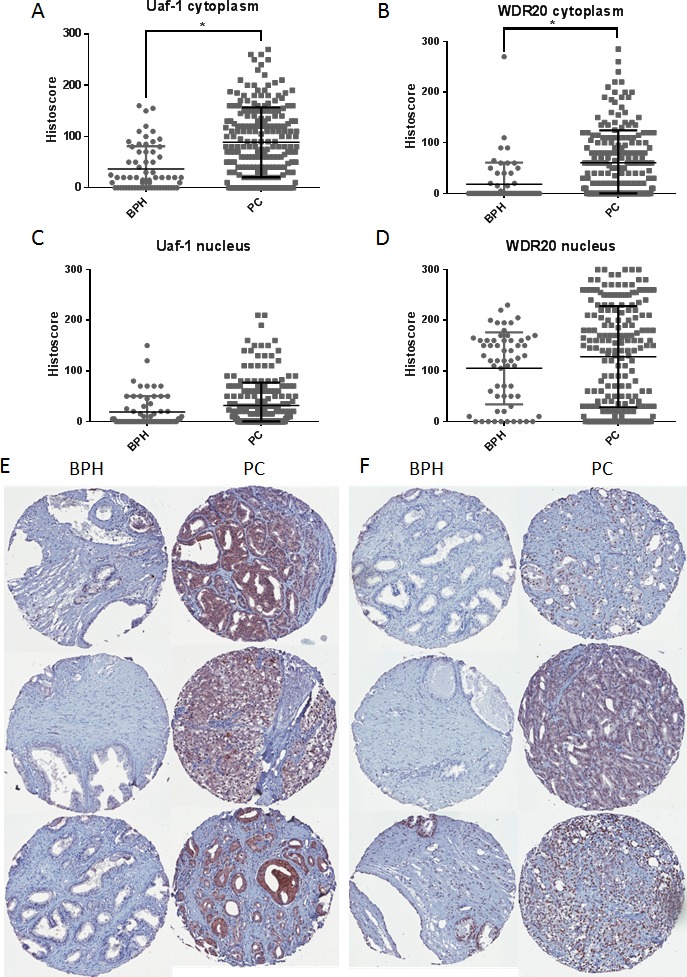
Protein levels of Uaf-1 and WDR20 are increased in PC **A.** Prostate TMA stained for Uaf-1. Data represents histoscore for cytoplasmic staining of 209 PC samples and 63 BPH samples +/− SEM. **B.** TMA stained for WDR20. Data represents histoscore for cytoplasmic staining of 195 PC samples and 59 BPH samples +/− SEM. **C.** Prostate TMA stained for Uaf-1. Data represents histoscore for nuclear staining of 209 PC samples and 63 BPH samples +/− SEM. **D.** TMA stained for WDR20. Data represents histoscore for nuclear staining of 195 PC samples and 59 BPH samples +/− SEM. **E.** Representative images of Uaf-1 staining in BPH and PC tissue. **F.** Representative images of WDR20 staining in BPH and PC tissue.

We previously demonstrated that Usp12 protein is overexpressed in PC compared to benign tissue [12]. We therefore predicted that expression of Uaf-1 and WDR20 would also be increased in PC. To test this we utilised a tissue microarray (TMA) containing samples from patients with benign prostatic hyperplasia (BPH) and PC. Staining for Uaf-1 and WDR20 revealed that both proteins were expressed at significantly higher levels in the cytoplasm in PC patients compared to benign controls (Figure 5A and 5B). A similar trend was observed in the nucleus (Figure 5C and 5D). Representative cores from each TMA are shown in Figure 5E and 5F. The increased level of expression of both proteins in PC underlines the importance of this complex during disease progression and highlights its potential role as a predictive marker and therapeutic target.

## DISCUSSION

The AR is a key driver of PC and as such is the major therapeutic target in advanced disease. However, treatments targeting AR invariably fail and result in the development of CRPC. Mechanisms that account for this include *AR* gene mutations, *AR* gene amplification, generation of constitutively active receptor splice variants and ligand-independent activation. Importantly, the AR is still expressed in CRPC and therefore it remains a viable therapeutic target [23].

The role of deubiquitination in AR regulation has not been extensively studied. Usp10 is known to enhance AR transcriptional activity [24, 25] and Usp22 and Usp26 have been found in complexes with AR [26, 27]. We recently identified the deubiquitinating enzyme Usp12 to be an AR co-activator [12]. Usp12 is known to function as part of a larger complex including the co-factors Uaf-1 and WDR20 without which Usp12 is catalytically inactive [16, 17]. In this report we investigated the role of these co-factors in regulating Usp12 and AR.

We established that Uaf-1 and WDR20 stabilise Usp12 at a protein level and that these cofactors work within a positive feedback loop at the mRNA and protein levels. We subsequently determined that Uaf-1 and WDR20 depletion alone is sufficient to destabilise AR and reduce its activity, highlighting the importance of this complex in AR regulation. Importantly, the effect of depleting these cofactors on AR activity was comparable between the PC cell lines representing both early and advanced disease. This suggests that AR is still regulated by the Usp12 complex in CRPC highlighting its potential as a therapeutic target. As Uaf-1 and WDR20 depletion reduces Usp12 levels their silencing has a comparable effect on the AR as the Usp12 depletion alone. Similar results have been seen in the case of Usp1/Uaf-1 where knockdown of either protein alone altered the ubiquitination status of Usp1 targets PCNA and FANCD2, in this example an epistatic relationship between Usp1 and Uaf-1 was observed [28]. It would be interesting to establish if similar principles could be applied to other Usp12 targets, for example, we have recently shown Usp12 depletion to sensitise PC cells to Akt inhibition [13]. It would therefore be worthwhile to investigate if individual depletion of Uaf-1 or WDR20 was also capable of achieving the same level of sensitisation.

Depletion of Usp12 co-factors decreased colony-forming ability in LNCaP cells and increased the numbers of apoptotic cells. Moreover, both Uaf-1 and WDR20 proteins were overexpressed in PC tissue. Taken together with their effect on AR stability and activity these results suggest that the Usp12 complex is worthy of further consideration as a future therapeutic target. In recent years, as more DUBs have been linked with cancer, DUB inhibitors have become the focus of extensive research. Preclinical success has been achieved with the small molecule b-AP15 that inhibits Usp14 and UCHL5, two DUBs associated with the 19S regulatory particle of the proteasome [29]. Several inhibitors of other DUBs including Usp7 have also been identified [30-33].

Many of the inhibitors developed against DUBs lack specificity, partly owing to the high degree of homology between DUB family members. Consequently targeting DUB complexes may allow the development of more targeted therapeutics. Work is already underway to develop inhibitors of the Usp1/Uaf-1 deubiquitinase complex. Early reports identified GW7647 and Pimozide as weak reversible non-competitive inhibitors, able to reverse cisplatin resistance in non-small cell lung cancer cells [21].

Recently, ML323 and its derivatives were identified as more potent and specific Usp1/Uaf-1 inhibitors. Authors suggested that ML323 specificity was achieved by targeting the DUB complex as opposed to the Usp1 active site. Again this drug reversed cisplatin resistance in non-small cell lung cancer cells [34, 35]. A further inhibitor of this complex reduced growth of primary human leukemia cells [36]. Overall work by these groups has confirmed the druggability of the Usp1/Uaf-1 complex and the advantages of complex targeting approach. We hypothesise that targeting the Usp12/Uaf-1/WDR20 complex in a similar manner would allow development of therapeutics with greater specificity and sensitivity than targeting Usp12 alone. We predict that this would be able to reduce AR activity and subsequently reduce proliferation of PC cells.

Interestingly, Usp12, Uaf-1 and WDR20 all contain a LXXLL-containing NR box- raising the possibility that this complex also interacts with other nuclear hormone receptors. It would therefore be worthwhile determining if depletion of the Usp12 complex is capable of reducing activity of other nuclear hormone receptors such as the oestrogen receptor. The cancer atlas within The Human Protein Atlas (www.proteinatlas.org) shows that Usp12 and Uaf-1 are expressed in breast and ovarian cancer tissues [37].

Finally, we have demonstrated that Uaf-1 and WDR20 are overexpressed in PC samples when compared to BPH. Taken together with our previous data demonstrating Usp12 overexpression in PC [12] this suggests that this complex could be a useful marker of disease. This is particularly pertinent as there is currently an acute lack of prognostic and predictive biomarkers in PC. Monitoring of PSA is currently used as part of PC diagnosis and as an indicator of recurrence. Although useful this method lacks sensitivity and as such has several limitations that can lead to over diagnosis and overtreatment of PC [38]. The identification of novel biomarkers is currently an important area of PC research.

In conclusion we have shown that Uaf-1 and WDR20 depletion reduces AR activity and stability, highlighting a further mechanism of AR regulation. We have also determined that these two cofactors are increased in PC and shown that depletion of these proteins alone increases apoptosis of PC cells. Overall this work suggests the Usp12 complex is worthy of future research to establish if it could be a therapeutic target or biomarker.

## MATERIALS AND METHODS

### Reagents and antibodies

Antibodies for anti-Usp12, anti-Flag, anti-WDR48 (Uaf-1) and anti-α-tubulin were purchased from Sigma-Aldrich. Other antibodies used were: anti-WDR20 (Abcam and Santa Cruz, 38K), anti-HA (Santa Cruz Biotechnology, Y-11) and anti-AR (Santa Cruz Biotechnology, N-20 and C-19).

### Cell culture

Tissue culture reagents were purchased from Sigma-Aldrich. LNCaP, CWR22Rv1, HEK293T and COS-7 cells were obtained from the American Type Culture Collection (Manassas, VA, USA). LNCaP-AI cells were derived in house as previously described [39]. VCaP cells were kindly donated by Professor Guido Jenster (Erasmus Medical Centre, Rotterdam). LNCaP-7b7 cells stably overexpressing pPSA-Luc vector were kindly donated by Professor Jan Trapman (Erasmus Medical Centre, Rotterdam) and cultured with the addition of 25 ng/ml Zeocin. LNCaP-AI PSA-Luc cells were generated in-house (K. Coffey and S. Walker, unpublished data). Cells were maintained in RPMI-1640 media with 2 mM L-glutamine (Invitrogen) supplemented with 10% (v/v) foetal calf serum at 37°C in 5% CO_2_. LNCaP-AI cells were maintained as above in RPMI-1640, 2 mM L-glutamine and 10% charcoal treated foetal calf serum, denoted steroid depleted media (SDM).

### Plasmids, transfection and siRNA gene silencing

pHA-Flag-WDR20 and pFlag-Uaf-1 were kind gifts from Professor Alan D'Andrea (Dana-Farber Cancer Institute, Boston). pFlag-Usp12, pPSA-Luc, pARE3-Luc, pCMV-β-gal and pFlag-His-AR were previously described [12]; [40]. All transfections were conducted using TransIT-LT1 reagent (MirusBiol) according to manufacturer's instructions.

Gene silencing was conducted using 25 nM siRNA, target sequences and knockdown efficiencies were as previously described [12]. Cells were reverse transfected using RNA iMAX (Invitrogen) according to manufacturer's instructions and incubated for 96 h unless otherwise indicated.

### Gene expression analysis

RNA was extracted using the EZ RNA isolation kit (Biological Industries) and quantified using the Nanodrop spectrophotometer (Thermo Scientific). cDNA synthesis and qRT-PCR was performed as previously described [41].

### Chromatin immunoprecipitation (ChIP)

ChIP was conducted as previously described [42] essentially following the protocol by Schmidt *et al*. [43]. LNCaP cells were subject to siRNA knockdown in steroid-depleted conditions for 96 h. 10 nM DHT was applied for the final 2 h prior to chromatin extraction. ChIP was conducted using anti-AR (Santa Cruz Biotechnology C-19) antibody or a negative IgG control. Data is presented as percentage input using the following formula: % Input = 100 × AE (amplification efficiency)*(CT adjusted input sample- CT immunoprecipitated sample). CT refers to cycle threshold. AREIII primer sequences were as follows F: TGGGACAACTTGCAAACCTG and R: CCAGAGTAGGTCTGTTTTCAATCCA.

### Flow cytometry

Apoptosis was assessed using the Annexin V assay (BD Biosciences) on the BD FACScan according to manufacturer's instructions. Assay was conducted 96 h post silencing and cells were stained with propidium iodide (PI) and anti-annexin V. During analysis cells were divided into quadrants representing healthy cells, necrotic cells and early and late apoptotic cells.

### Immunoprecipitation

LNCaP cells were seeded at a density of 1×10^6^ cells per 90 mm dish, incubated for 96 h and immunoprecipitated as previously described [12]. Alternatively, HEK293T cells were seeded at a density of 5×10^5^ per 90 mm dish. After 24 h cells were transfected with 1 μg of each of the following plasmids in varying combinations: pFlag-AR, pFlag-Usp12, pFlag-Uaf-1 and pHA-Flag-WDR20. Cells were incubated for a total of 48 h prior to immunoprecipitation.

### Luciferase reporter assay

For overexpression studies HEK293T cells were seeded to a 24 well plate in SDM and 24 h later transfected with 150 ng pARE3-luc, 50 ng pCMV-β-gal, 10 ng pFlag-His-AR and varying combinations of pFlag-Usp12, pFlag-Uaf-1 and pHA-Flag-WDR20 at 10 ng each. All reactions were balanced with pCMV empty vector. Cells were treated with 10 nM DHT 24 h later and incubated for a final 24 h prior to lysis in 1x Reporter Lysis Buffer (Promega). Lysis solution was mixed with Luciferin reagent (Promega) according to manufacturer's instructions and luciferase counts determined using the FLUOstar Omega microplate reader (BMG LABTECH). Results were normalised to β-galactosidase activity.

For knockdown studies LNCaP-7b7 or LNCaP-AI PSA-Luc cells were subject to 96 h siRNA silencing as indicated. LNCaP-7b7 cells were grown in SDM for 72 h followed by 24 h stimulation with 10 nM DHT. LNCaP-AI PSA-Luc cells were cultured in SDM for 96 h. Luciferase activity was determined as above and results were normalised to cell density determined by live cell imaging with IncuCyte ZOOM (Essen Bioscience) immediately prior to lysis.

### Proliferation assays

Proliferation was determined by cell counting following trypan blue exclusion assay after 96 h of continuous siRNA silencing. Percentage cell survival compared to scrambled (SCR) control was determined.

### Colony formation assays

LNCaP cells were reverse transfected with siRNA and incubated for 72 h. Cells were reseeded over a range of cell densities and incubated for 14 days prior to fixing with Carnoy's Fixative and staining with crystal violet. Colonies were counted and the fold change in colony forming ability compared to SCR control.

### Immunohistochemistry

Immunohistochemistry was conducted as previously described using 0.01M Citrate buffer at pH 6.0 for antigen retrieval [12] with anti-Uaf-1 antibody (Sigma-Aldrich) at 1.25μg/μl or anti-WDR20 (Abcam) at 0.04 μg/μl. Tissue microarrays (TMAs) were independently scored by two scorers using the 0-300 Histoscore [44]. Briefly, percentage and intensity of staining for positive cells was estimated (0, 1, 2, 3) using the following equation Histoscore = (% of cells with low level positivity scored as 1 × 1) + (% of cells with medium level positivity scored as 2 × 2) + (% of cells with high level positivity scored as 3 × 3). Histoscore was used to independently score cytoplasmic and nuclear staining.

### Statistical analysis

All data was first tested for Gaussian distribution. In all cases data was found to be parametric and t-test was used for further analysis. p values of below 0.05 were considered statistically significant and marked with an asterisk (*). Immunohistopathological analysis was performed using non-parametric statistics with previously mentioned thresholds applied to p value.

## SUPPLEMENTARY MATERIAL FIGURE


