# Supplementary material for: Ubiquitin-specific protease 12 interacting partners Uaf-1 and WDR20 are potential therapeutic targets in prostate cancer
Source: Oncotarget. 2015 Oct 10;6(35):37724–36. doi: 10.18632/oncotarget.6075 (PMC4741960; doi:10.18632/oncotarget.6075)
Supplement: Supplementary file 1 [file oncotarget-06-37724-s001.pdf]

Ubiquitin-specific protease 12 interacting partners Uaf-1 and WDR20 are potential therapeutic targets in prostate cancer

Supplementary Material

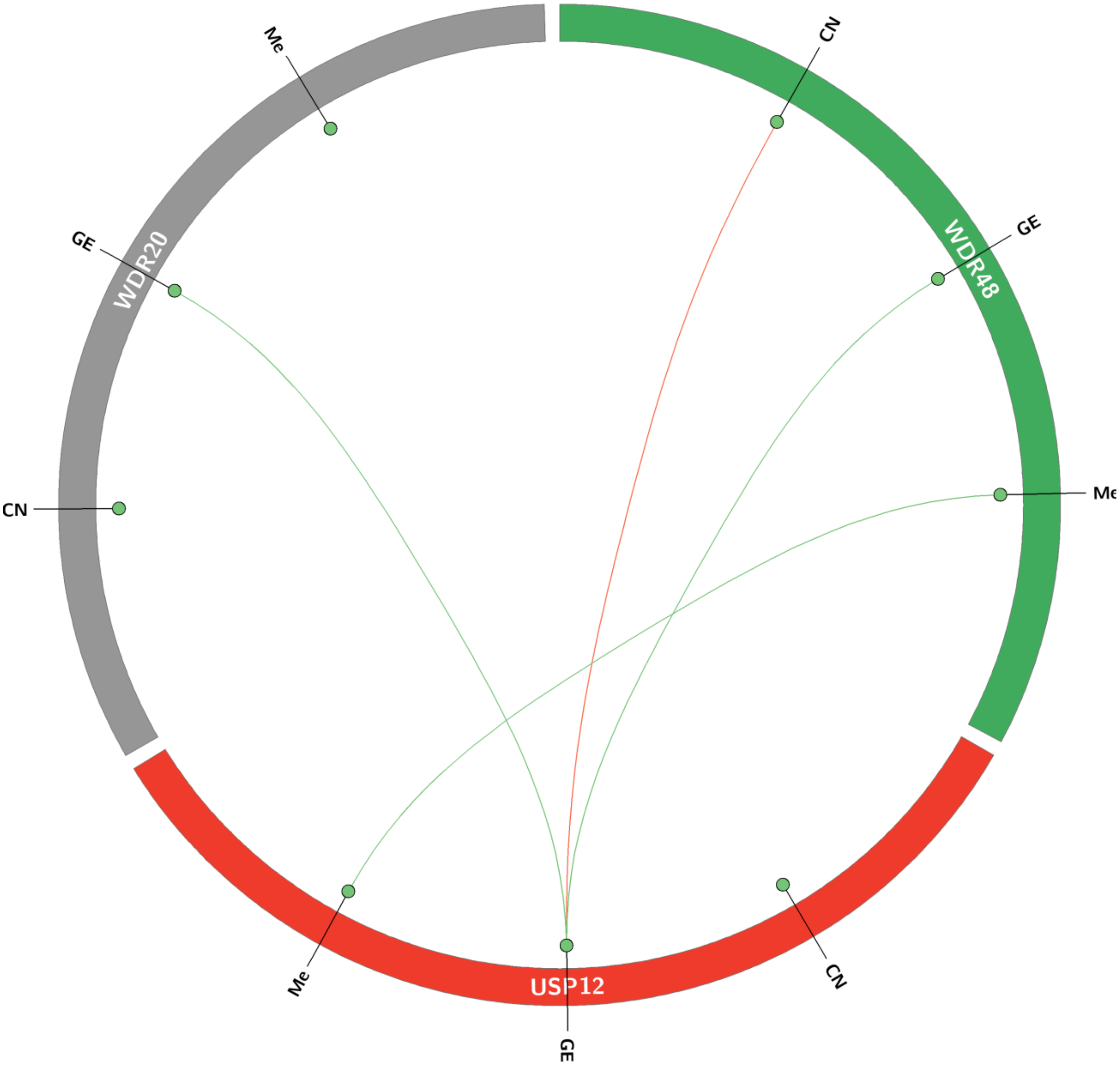

Supplementary figure 1.

ZODIAC analysis of the TCGA dataset for correlation between Usp12, Uaf-1 (WDR48) and WDR20 gene expression (GE), copy number (CN) and methylation (Me) levels. Positive correlations are marked in green and negative correlations in red.
